# Cell biological insights into human STING variants

**DOI:** 10.1247/csf.25020

**Published:** 2025-05-14

**Authors:** Shogo Koide, Eisuke Yumoto, Jun Nakayama, Shigeki Higashiyama, Yoshihiko Kuchitsu, Tomohiko Taguchi

**Affiliations:** 1 Laboratory of Organelle Pathophysiology, Department of Integrative Life Sciences, Graduate School of Life Sciences, Tohoku University, Aoba-ku, Sendai, Japan; 2 Department of Oncogenesis and Growth Regulation, Osaka International Cancer Institute, Chuo-ku, Osaka, Japan

**Keywords:** innate immunity, STING variants, COPA syndrome, membrane traffic, the Golgi

## Abstract

Stimulator of interferon genes (STING) is an endoplasmic reticulum (ER)-localized transmembrane protein. STING induces type I interferon and inflammatory responses against a variety of double-stranded DNA (dsDNA) viruses, which is critical for limiting their infection and replication. In certain settings where self-DNAs (genomic or mitochondrial DNA) emerge in the cytosol or when intracellular membrane traffic is impaired, STING becomes activated and triggers inflammation, which may contribute to the pathogenesis of various autoinflammatory and neurodegenerative diseases, including COPA syndrome and Parkinson’s disease. The human STING gene exhibits genetic heterogeneity with R232, HAQ (R71H-G230A-R293Q), and H232 being the most common variants, along with population stratification. A very recent study has shown that HAQ, not R232 or H232, mediates complete clinical protection in the pathogenesis of COPA syndrome. These results reveal, for the first time, the distinct activities of the major variants in the context of the pathogenesis of autoinflammatory diseases. Besides these major variants, there exist minor pathogenic STING variants that cause an autoinflammatory disease called STING-associated vasculopathy with onset in infancy (SAVI). This review summarizes recent insights into human STING variants and their inflammatory activities.

## Graphical Abstract

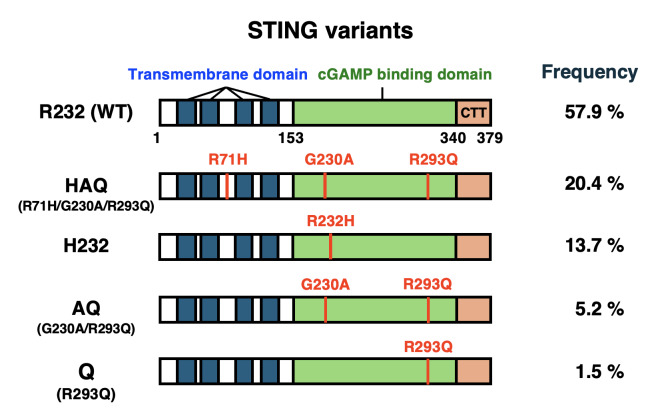

## cGAS-STING Innate Immune Signalling Pathway

The detection of microbial pathogens with nucleic acid sensors is one of the central strategies in innate immunity (Palm and Medzhitov, 2009; Takeuchi and Akira, 2010). Cyclic GMP-AMP synthase (cGAS) is a sensor for dsDNA in the cytosol (Sun *et al.*, 2013). cGAS synthesizes 2'3'-cyclic GMP-AMP (cGAMP) with ATP and GTP (Wu *et al.*, 2013), which induces the type I interferon and inflammatory responses through the cGAMP sensor STING, an ER-localized transmembrane protein (Ishikawa *et al.*, 2009; Ishikawa and Barber, 2008) [also known as MITA (Zhong *et al.*, 2008), MPYS (Jin *et al.*, 2008), ERIS (Sun *et al.*, 2009), or TMEM173].

After binding to cGAMP, STING translocates to the trans-Golgi network (TGN) through the Golgi where STING undergoes palmitoylation at Cys88 and Cys91 (Hansen *et al.*, 2018; Matsumoto *et al.*, 2023; Mukai *et al.*, 2016). Palmitoylated STING forms clusters in cholesterol- and sphingomyelin-rich lipid microdomains in the TGN, promoting TBK1 autophosphorylation and activation on STING (Kemmoku *et al.*, 2022, 2024; Takahashi *et al.*, 2021). The activated TBK1 then phosphorylates the transcription factor interferon regulatory factor 3 (IRF3) (Tanaka and Chen, 2012). Phosphorylated IRF3 by TBK1 dimerizes and translocates into the nucleus to induce transcription of genes that encode type I interferons such as interferon-β (IFN-β). STING also induces proinflammatory response via the activation of Nuclear Factor Kappa B (NF-κB), which is mediated by TBK1 and IκB kinase epsilon (IKKε) (Balka *et al.*, 2020; Venkatraman *et al.*, 2024), and by the Linear Ubiquitin Chain Assembly Complex (LUBAC) (Fischer *et al.*, 2025).

STING further moves from the TGN to recycling endosomes (REs) where STING undergoes K63-linked polyubiquitination at Lys288 (Kuchitsu *et al.*, 2023). This ubiquitination is required for the endosomal sorting complexes required for transport (ESCRT)-driven lysosomal microautophagic degradation of STING (Kuchitsu *et al.*, 2023; Kuchitsu and Taguchi, 2024; Shoji *et al.*, 2025). The impaired post-Golgi membrane traffic of activated STING or dysfunction of the ESCRT complex results in the prolonged inflammatory signals, which may be associated with a variety of autoinflammatory and neurodegenerative diseases including amyotrophic lateral sclerosis (ALS) (McCauley *et al.*, 2020) and hereditary spastic paraplegia (HSP) (Farazi Fard *et al.*, 2019; Gu *et al.*, 2020; Lin *et al.*, 2019; Zivony-Elboum *et al.*, 2012).

**Fig. 1:**
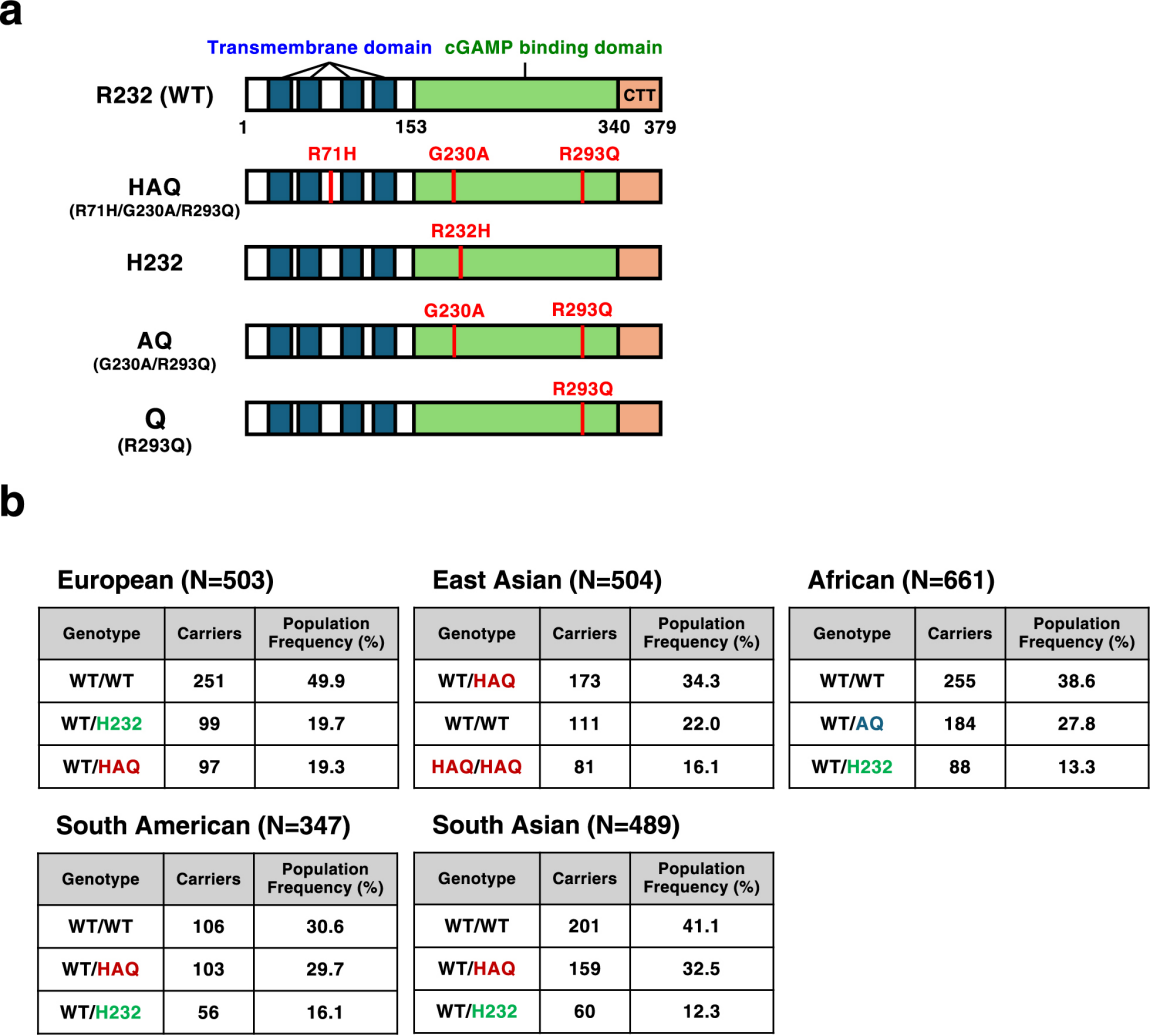
Major human STING variants a, Domain organization of human STING and positions of amino acid substitutions in its variants: transmembrane domain (blue), the cGAMP binding region (green), and the C-terminal tail (CTT) (beige). b, STING genotypes found in the five ethnic groups in the 1000 Genomes Project (phase III) (Patel *et al.*, 2017).

The human STING gene holds genetic heterogeneity with R232, HAQ (R71H-G230A-R293Q), and H232 being the most common variants, and population stratification (Patel *et al.*, 2017) (Fig. 1a). R232 is defined as wild-type human STING. A very recent study has shown that HAQ, not R232 or H232, mediates completely clinical protection in the pathogenesis of COPA syndrome (Simchoni *et al.*, 2025). Besides these major variants, there exist minor pathogenic STING variants that cause STING-associated vasculopathy with onset in infancy (SAVI) (Frémond *et al.*, 2021). SAVI variants activate TBK1 at the TGN without dsDNA/cGAMP stimulation (Jeremiah *et al.*, 2014; Kemmoku *et al.*, 2022; Liu *et al.*, 2014; Mukai *et al.*, 2016; Ogawa *et al.*, 2018), and are thus regarded as constitutively active forms. This review summarizes recent insights into human STING variants, and discusses how the intracellular membrane traffic is associated with their inflammatory activities.

## Major Human STING Variants

Human STING protein is encoded by the *STING1* gene (Gene ID: 340061). Several major allelic variants have been reported, known as R232 (*rs1131769*), H232, and HAQ (R71H, G230A, R293Q) where R71H (*rs11554776*), G230A (*rs78233829*), and R293Q (*rs7380824*) are in linkage disequilibrium (Fig. 1a). These variants distribute differently among distinct ethnic populations (Fig. 1b). In Europeans, population frequency of R232/R232 is 49.9% and this genotype is the most dominant. Because of the dominance of R232, R232 is commonly regarded as wild-type STING. In East Asians, the allele frequency of HAQ is relatively high and the most dominant genotype is HAQ/R232 (population frequency 34.3%). In contrast, HAQ/HAQ was not found among 661 individuals examined in Africans (Patel *et al.*, 2017).

Different activities of these major STING variants towards 2'3'-cGAMP have been reported *in vitro* cell-based assays. In HEK293T cells transiently expressing the individual variants, R232, HAQ, or H232 can equally induce the interferon response (Yi *et al.*, 2013). In contrast, in THP1 cells stably expressing the individual variants, R232 and HAQ, not H232, can induce the interferon response (Froechlich *et al.*, 2023). The inability of H232 to induce the interferon response towards 2'3'-cGAMP is also reported in L929 cells stably expressing H232 (Zhang *et al.*, 2013). A biochemical study with diﬀerential scanning fluorimetry shows that H232 has less affinity to 2'3'-cGAMP than R232, supporting that H232 is a hypomorphic variant for 2'3'-cGAMP stimulation (Vavřina *et al.*, 2021).

There have been a few *ex vivo* studies using primary cells. B cells isolated from HAQ/HAQ carriers express a low level of STING (HAQ) and do not respond to 2'3'-cGAMP (Patel *et al.*, 2017). Peripheral blood mononuclear cells (PBMC) from HAQ/HAQ carriers show less interferon response in response to 2'3'-cGAMP, compared to PBMC from R232/R232 (Ruiz-Moreno *et al.*, 2018).

## A New Insight from COPA Syndrome

**Fig. 2:**
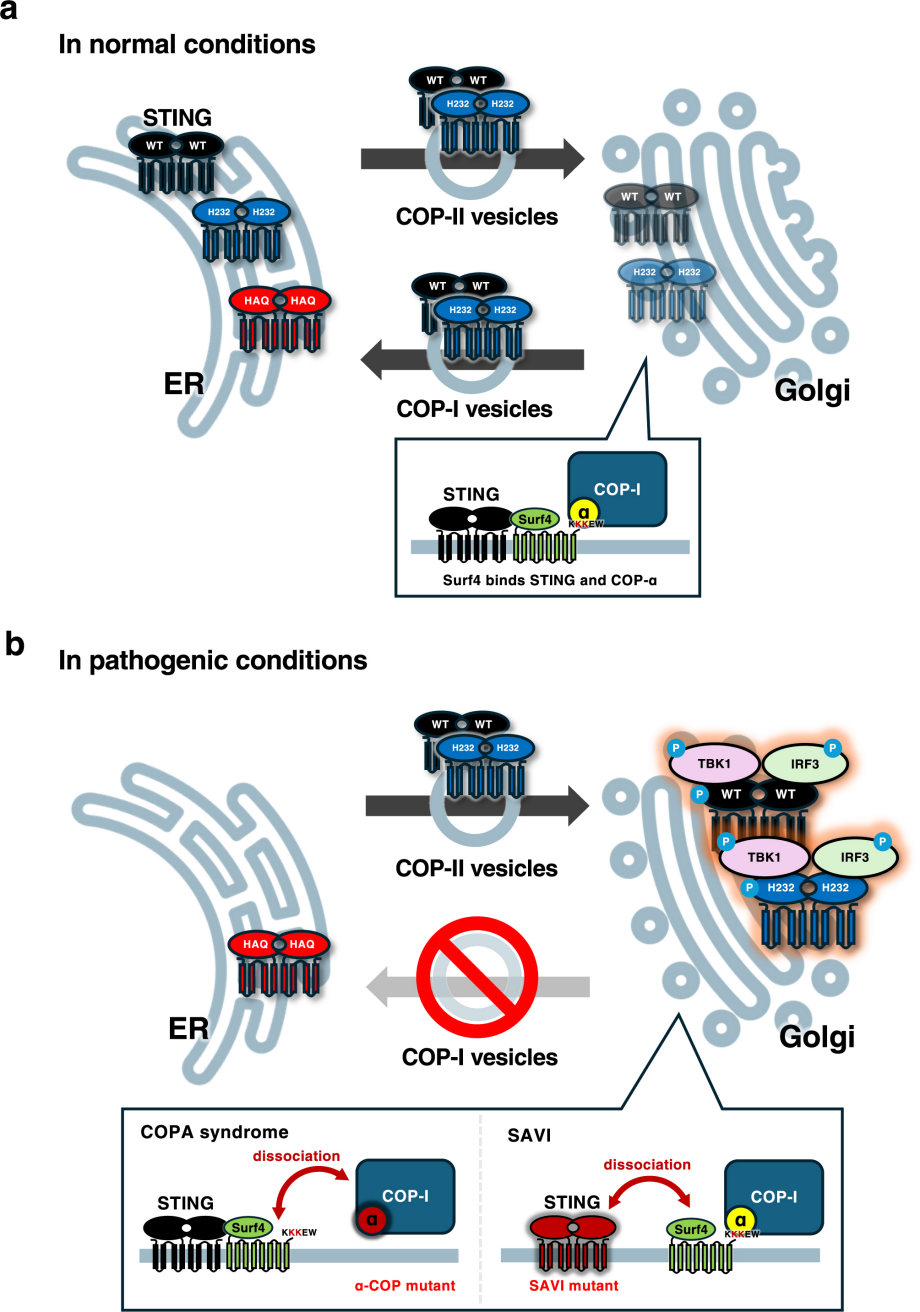
Activation of WT and H232 STING in COPA disease In normal conditions, WT (R232) and H232 translocate to the Golgi with the COP-II machinery, whereas HAQ remains localized at the ER. WT (R232) and H232 are then retrieved back to the ER with the COP-I machinery, ensuring their predominant localizations at the ER. In the conditions where pathogenic α-COP is expressed, WT (R232) and H232 cannot be retrieved back to the ER, leading to their activations at the TGN. SAVI-STINGs have a reduced affinity to Surf4, making them poor cargos for the COP-I transport.

Coat protein complex I (COP-I) mediates the retrograde transport from the Golgi to the ER. Mutation of the *COPA* gene, encoding one of the COP-I subunits (α-COP), causes an immune dysregulatory disease known as COPA syndrome (Watkin *et al.*, 2015). The patients have high-titer autoantibodies, and develop inflammatory arthritis and interstitial lung disease (Watkin *et al.*, 2015). We and others found that mis-trafficking of STING to the Golgi is the main cause of the inflammation in COPA syndrome (Deng *et al.*, 2020; Kato *et al.*, 2021; Lepelley *et al.*, 2020; Mukai *et al.*, 2021; Steiner *et al.*, 2022). Expression of pathogenic α-COP variants in cGAS-knockout cells, as in control cells, relocates STING from the ER and activates STING, suggesting that cGAS/cGAMP is not essential for STING traffic out of the ER to the Golgi, nor for STING activation (Mukai *et al.*, 2021). α-COP binds C-terminal di-lysine motifs of its cargo proteins, such as KKXX and KXKXX (Cosson and Letourneur, 1994; Letourneur *et al.*, 1994; Ma and Goldberg, 2013). As STING does not possess these motifs at its C-terminus, we reason the presence of adapter protein(s) that mediates the interaction of STING and α-COP, and find that Surf4, a transmembrane protein with KKEW sequence at its C-terminus, functions as the tether (Mukai *et al.*, 2021). Based on these observations, we provide a model that explains the spontaneous STING activation in the presence of pathogenic α-COP variants: STING constantly circulates between the ER and the Golgi in unstimulated conditions. Anterograde traffic from the ER to the Golgi is mediated by the COP-II transport system, whereas retrograde traffic from the Golgi to the ER is mediated by the COP-I transport system (Fig. 2a). When the retrograde transport is impaired by the expression of pathogenic α-COP variants with a reduced affinity for Surf4, STING becomes accumulated at the Golgi and eventually reaches the TGN, leading to the activation of STING (Fig. 2b).

Intriguingly, COPA syndrome, is nonpenetrant in ~20% of individuals, with no known mediators of protection. A very recent study shows that the HAQ variant mediates complete clinical protection (Simchoni *et al.*, 2025). In the study, 35 individuals with COPA mutations (26 affected patients and 9 unaffected carriers) are sequenced, and HAQ is found to be co-segregated with clinical nonpenetrance. Exome sequencing identifies only the mutations comprising HAQ as variants shared by unaffected carriers and absent in patients. Expression of HAQ in patient cells rescues the molecular phenotype of COPA syndrome. Cell biological analysis of cells with knockdown of *COPA*, shows that R232 and H232 translocate to the Golgi, whereas HAQ remains localized at the ER. These results indicate the distinct trafficking ability of these three major variants in unstimulated conditions: HAQ may have a reduced affinity for the COP-II machinery than R232 and H232 in the absence of cGAMP (Fig. 2a and b).

Of note, the 9 unaffected carriers have one allele of HAQ. The dominant ability of HAQ over R232 in quenching the interferon response may be due to the ability of HAQ to form a heterodimer with R232 (Simchoni *et al.*, 2025).

## Minor Pathogenic Human STING Variants

**Fig. 3:**
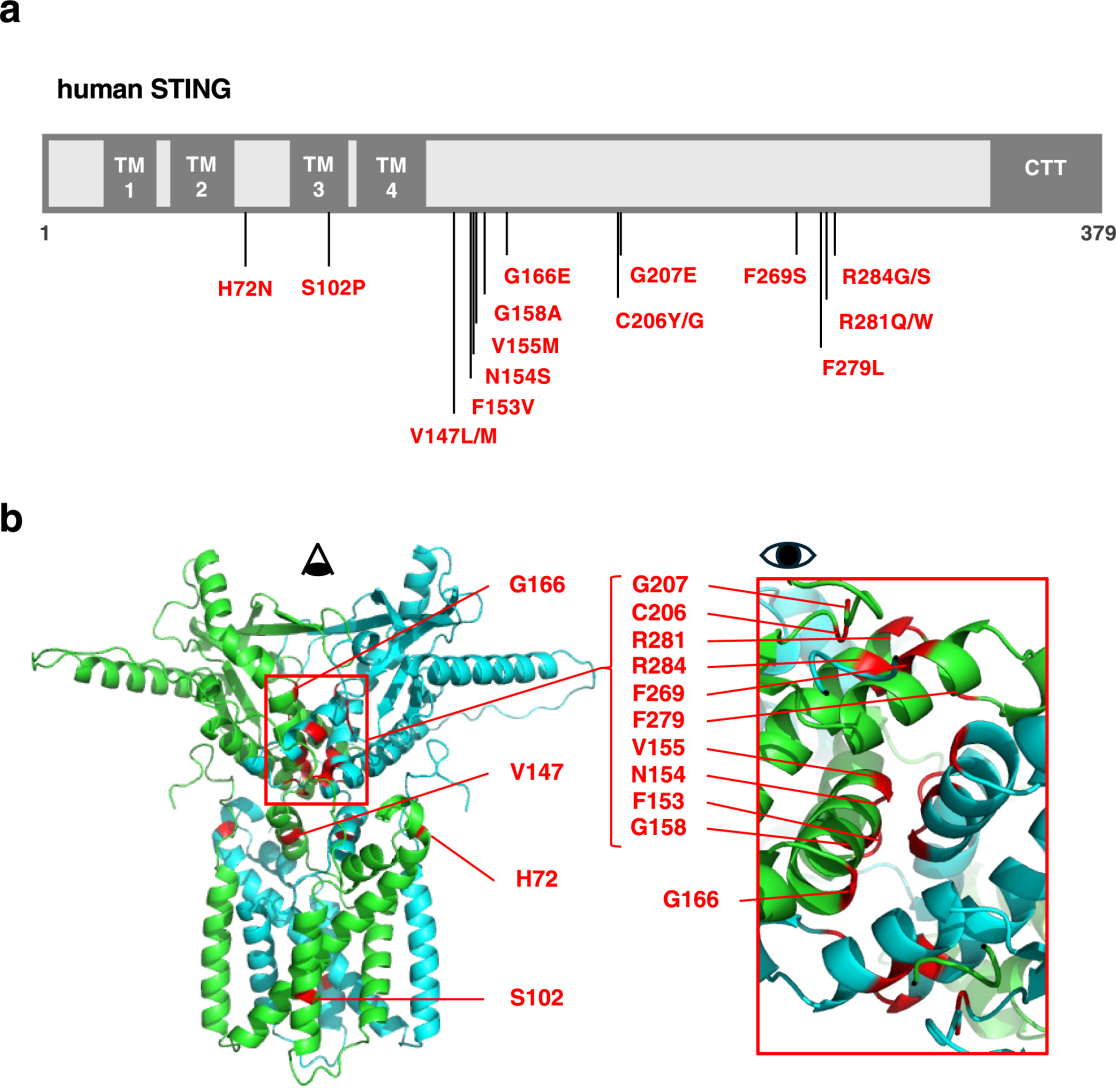
Amino acid substitutions in SAVI-STING variants a, The vertical lines indicate the positions of the amino acid substitutions in SAVI-STING. b, The SAVI mutations (red) are superimposed on the three-dimensional structure of a wild-type STING dimer modeled using AlphaFold3.

STING-associated vasculopathy with onset in infancy (SAVI) is a systemic inflammatory disorder that primarily affects the skin, blood vessels, and lungs (Frémond *et al.*, 2021). Multiple STING mutations have been identified in SAVI patients, including H72N (Lin *et al.*, 2021), S102P/F279L (Seo *et al.*, 2017), V147L (Liu *et al.*, 2014), V147M (Munoz *et al.*, 2015), F153V (Lin *et al.*, 2021), N154S (Liu *et al.*, 2014), V155M (Jeremiah *et al.*, 2014; Liu *et al.*, 2014), G158A (Lin *et al.*, 2021), G166E (König *et al.*, 2017), C206Y (Melki *et al.*, 2017), C206G (Manoussakis *et al.*, 2017), G207E (Keskitalo *et al.*, 2019), F269S (Valeri *et al.*, 2024), R281Q (Melki *et al.*, 2017), R281W (Lin *et al.*, 2020) and R284G (Melki *et al.*, 2017) and R284S (Konno *et al.*, 2018; Saldanha *et al.*, 2018) (Fig. 3a). Of note, most of the SAVI mutations are found in the dimer interface (Fig. 3b).

These SAVI variants constitutively activate the inflammatory response in a cGAMP-independent manner. They do not show the ER-localization and reach the TGN to activate TBK1 (Jeremiah *et al.*, 2014; Kemmoku *et al.*, 2022; Liu *et al.*, 2014; Mukai *et al.*, 2016; Ogawa *et al.*, 2018). Intriguingly, some of SAVI STINGs show a reduced affinity to Surf4, a cargo receptor essential for STING retrieval from the Golgi to the ER (Mukai *et al.*, 2021). The reduced interaction may impair the STING traffic back to the ER, resulting in the accumulation of SAVI variants at the Golgi and their activation at the TGN (Fig. 2b).

As aforementioned, V155M variant localizes at the Golgi and the post-Golgi compartments (Jeremiah *et al.*, 2014). In contrast, a STING variant having V155M and the HAQ mutations simultaneously, localizes at the ER and loses the ability to induce the type I interferon response (Cerboni *et al.*, 2017). These results enforce the notice from the COPA syndrome that the HAQ mutation may decrease the binding affinity of STING to the COP-II machinery (Simchoni *et al.*, 2025).

SAVI mouse models have been generated with N153S or V154M mutation on murine STING, corresponding to human N154S or V155M mutation, respectively. These mice exhibit some of the expected disease outcomes including spontaneous lung inflammation, T cell cytopenia, and elevated expression of type I interferon-stimulated genes in multiple tissues (Motwani *et al.*, 2019; Warner *et al.*, 2017), demonstrating that SAVI mutations sufficiently drive the inflammatory responses also in mice. Importantly, SAVI mutations in mice without a functional type I interferon response still mediate disease progression. T-cell receptor β chain (*Tcrb*)^–/–^ STING N153S mice that lack αβ T cells only have mild lung disease or no disease at all, and recombination-activating gene 1 (*Rag1*)^–/–^ STING N153S mice exhibit no histologic sign of lung inflammation, suggesting that T cells play a dominant role in promoting lung disease (Luksch *et al.*, 2019). SAVI phenotype of STING V154M mice is not rescued by IRF3 or IFN-α/β receptor deficiency (Motwani *et al.*, 2019). A recent study shows that mice heterozygously having N153S and HAQ allele exhibit an attenuated inflammatory phenotype (Aybar-Torres *et al.*, 2024). It may be interesting to examine whether HAQ variant can form heterocomplex with N153S, retaining N153S in the ER and preventing spontaneous activation of N153S at the TGN. Of note, the SAVI patient with S102 and F279L mutations, have one HAQ allele without S102P and F279L mutations (Seo *et al.*, 2017). Whether HAQ variant can form heterocomplex with S102/F279L remains to be elucidated.

## Perspectives

Recent studies unveil the close association of STING to various autoinflammatory and neurodegenerative diseases including Parkinson’s disease (Sliter *et al.*, 2018), ALS (McCauley *et al.*, 2020; Yu *et al.*, 2020), Niemann-Pick disease type C (Chu *et al.*, 2021), and aging-related diseases (Gulen *et al.*, 2023; Hamann *et al.*, 2019). Aged Parkin-knockout mice expressing a proofreading-defective mitochondrial DNA polymerase γ is a model of Parkinson’s disease, an age-related degenerative brain condition. These mice lose dopaminergic neurons from the substantia nigra pars compacta and show the motor defect. These pathological features are rescued by loss of STING, indicating that cytosolic DNA released from mitochondria, activates cGAS-STING signalling pathway, leading to inflammation and neuronal cell death (Sliter *et al.*, 2018). Cytosolic DNA released from mitochondria is also indicated to facilitate aging-related symptoms such as neurodegeneration and cognitive decline (Gulen *et al.*, 2023). Of note, a STING Q293 variant (R293Q) is associated with protection from combined aging-related diseases, cardiovascular disease and chronic lung diseases (Hamann *et al.*, 2019).

Given that HAQ variant, one of the STING major variants, has the ability to cancel the pathogenesis of COPA syndrome (Simchoni *et al.*, 2025), onset and severity of other STING-mediated diseases can be influenced by STING major variants. To reveal the causal link between STING major variants and the pathogenesis of STING-mediated diseases, it will be critical to read *STING1* sequence in the patient and its pedigree. Generating knock-in mouse with human STING major variants will benefit the *in vivo* examination of the link. Consideration of the skewed ethnical distribution of STING major variants (Fig. 1b) may help identify STING-mediated endemic autoinflammatory and infectious diseases.

A recent study on COPA syndrome suggests that HAQ variant is resistant to the translocation from the ER to the Golgi (Simchoni *et al.*, 2025). However, its underlying mechanism is unclear. The determination of three-dimensional structure of HAQ and the comparison with that of R232 or H232 may help understand the ER retention mechanism. HAQ can form a heterodimer with R232, which may explain the dominant ability of HAQ over R232 in quenching the interferon response (Simchoni *et al.*, 2025). To examine whether STING variants can form heterodimer with each other and how the individual heterodimers are trafficked out from the ER will also be essential for understanding the distinct activation properties of STING variants.

Cancer immunotherapy has transformed the treatment of cancer, with a success of immune checkpoint inhibitors of programmed cell death 1 and its ligand (Chamoto *et al.*, 2023). The search for other immune regulators has been extended to innate immune signalling, which is expected to enhance tumour immunogenicity. The cGAS-STING pathway, in particular in dendritic cells, has emerged as a critical intrinsic tumour-detecting mechanism (Barber, 2015; Klarquist *et al.*, 2014; Woo *et al.*, 2014). Various small chemicals including cGAMP derivatives have been developed, and they show a significant anti-tumour activity in mice (Wang *et al.*, 2024). In contrast, the activation of STING in tumour cells results in different outcomes. It can be detrimental for survival of tumour cells (Kitajima *et al.*, 2019; Murayama *et al.*, 2024; Yan *et al.*, 2023) or beneficial for their metastasis (Chen *et al.*, 2016; Hu *et al.*, 2023). Given the impact of STING activation on anti-tumour immunity, it is critical to validate the degree of contribution of individual STING major variants into anti-tumour immunity and tumour progression. As discussed, generating knock-in mouse with human STING major variants will benefit the *in vivo* validation.

## Author Contributions

S.K. and E.Y. gathered the information over the review’s topics and prepared the figures. J.N. and S.H. discussed the impact of STING variants on cancer progression. Y.K. and T.T. conceptualized the layout of the topics and wrote the review.

## Conflict of Interest

The authors declare no competing financial interests.
